# Selected *Schizosaccharomyces pombe* Strains Have Characteristics That Are Beneficial for Winemaking

**DOI:** 10.1371/journal.pone.0151102

**Published:** 2016-03-23

**Authors:** Ángel Benito, Daniel Jeffares, Felipe Palomero, Fernando Calderón, Feng-Yan Bai, Jürg Bähler, Santiago Benito

**Affiliations:** 1 Department of Chemistry and Food Technology, Polytechnic University of Madrid, Madrid, Spain; 2 Research Department of Genetics, Evolution and Environment, University College London, London, United Kingdom; 3 State Key Laboratory of Mycology, Institute of Microbiology, Chinese Academy of Sciences, Beijing, China; University of Cambridge, UNITED KINGDOM

## Abstract

At present, wine is generally produced using *Saccharomyces* yeast followed by *Oenococus* bacteria to complete malolactic fermentation. This method has some unsolved problems, such as the management of highly acidic musts and the production of potentially toxic products including biogenic amines and ethyl carbamate. Here we explore the potential of the fission yeast *Schizosaccharomyces pombe* to solve these problems. We characterise an extensive worldwide collection of *S*. *pombe* strains according to classic biochemical parameters of oenological interest. We identify three genetically different *S*. *pombe* strains that appear suitable for winemaking. These strains compare favourably to standard *Saccharomyces cerevisiae* winemaking strains, in that they perform effective malic acid deacidification and significantly reduce levels of biogenic amines and ethyl carbamate precursors without the need for any secondary bacterial malolactic fermentation. These findings indicate that the use of certain *S*. *pombe* strains could be advantageous for winemaking in regions where malic acid is problematic, and these strains also show superior performance with respect to food safety.

## Introduction

Several research teams are studying the winemaking potential of non-*Saccharomyces* yeast strains [1]. For example, fission yeasts from the genus *Schizosaccharomyces* show more rapid malic acid deacidification, by converting malic acid to ethanol and CO_2_ [2–4]. *Schizosaccharomyces pombe* possesses several remarkable metabolic properties that may be useful in modern quality winemaking [2,5–6], including a malic dehydrogenase activity, high autolytic polysaccharides release [7], ability of gluconic acid reduction [8–11], urease activity [12,13], elevated production of pyruvic acid and colour improvement [14,15], as well as low production of biogenic amines and ethyl carbamate [6,16].

However, *S*. *pombe* type strains are not currently employed because of specific off-flavours commonly associated with their metabolism. Indeed, *Schizosaccharomyces* strains are commonly isolated from wines suffering from strong organoleptic and chemical deviations including the appearance of acetic acid, acetaldehyde, acetoin and ethyl acetate [17–21]. These undesirable properties of the commonly-used strains have been assumed to be general properties of the species, and only one commercial strain of *S*. *pombe* is currently available on the market [22]. The exploration of the winemaking properties of other *S*. *pombe* strains has generally been overlooked. This omission partly reflects the absence of any specific processes to select strains that are appropriate for winemaking [5], and difficulties with isolating *S*. *pombe* from environmental samples [23]. Given this situation, it has been difficult to obtain a representative strain collection of this species. However, guided by a recent analysis of genetic and phenotypic diversity of 161 *S*. *pombe* strains [24], we can now conduct a more extensive survey of the utility of this species for winemaking.

The present study examines the winemaking potential of 75 *S*. *pombe* strains that are available from different microorganism type culture collections, or have been collected by us [5,25]. We apply classic selection parameters used in traditional fermentative *Saccharomyces* yeasts, along with more advanced and specific parameters for *S*. *pombe*. The results indicate that at least three *S*. *pombe* strains can potentially provide a viable and attractive alternative to the application of genetically modified *Saccharomyces* species [26–29] whose use is restricted in food industry by European legislation [30].

## Materials and Methods

### Microorganisms

The following yeasts were used for the experimental fermentations: 75 *S*. *pombe* strains, including the non-clonal 54 strains recently described [24], the three mating types of the standard laboratory strain: Leupold’s 972 (h^−^), 975 (h^+^) and 968 (h^90^) (JB22, JB32 and JB50), strain V1 collected by us [5], and 17 strains collected from traditional Chinese breweries by Feng-Yan Bai (unpublished). The following *S*. *cerevisiae* strains were used as controls: 87 and 88 from the Spanish Type Culture collection, which have been used in previous studies. Data for all strains are provided in Table 1.

**Table 1:** *Schizosaccharomyces pombe* yeast strains used in the experiments.

Bähler laboratory strain name	strain ID(s)	location collected	substrate	date collected	reference
JB4	CBS5557;CCY44-6-1;CBS10393;DBVPG6275;JCM8262;NBRC1608;NCYC683	Spain	Listan grapes	ND	NA
JB22	Leupolds 972 (h-);CBS10395;NCYC1430	France	rotten grapes	1947	NA
JB32	Leupolds 975 (h+)				NA
JB50	Leupolds 968 (h90)	France	ND	ND	NA
JB758	NOTT30;NCYC936;CBS10394	Sri Lanka	fermenting toddy	1979	PMID:25665008
JB759	NOTT33;Y0036;CBS10498	South Africa	beverage	ND	PMID:25665008
JB760	NOTT50;DBVPG2812	Italy (Sicily)	grape must treated with SO2	1966	PMID:25665008
JB762	NOTT75;CBS358;DBVPG6374	ND	ND	1987	PMID:25665008
JB837	NOTT1;UWOPS92.229.4	Mexico	Tequilla	1992	PMID:25665008
JB838	NOTT2;UWOPS94.422.2	Mexico	Tequilla	1994	PMID:25665008
JB840	NOTT4;UFMGR435;CBS10458	Brazil (Aracaju)	frozen pulp of Eugenia uniflora ("pitanga", Myrtaceae)	1999	PMID:25665008
JB841	NOTT5;UFMGA1263;CBS10469	Brazil (Vicosa)	must of Brazilian cachaça	1996	PMID:25665008
JB842	NOTT6;UFMGA602;CBS10460	Brazil (Belo Horizonte)	must of Brazilian cachaça	1996	PMID:25665008
JB845	NOTT9;UFMGR434;CBS10476	Brazil (Aracaju)	frozen pulp of Eugenia uniflora ("pitanga", Myrtaceae)	1999	PMID:25665008
JB846	NOTT10;UFMGA826;CBS10465	Brazil (Belo Horizonte)	must of Brazilian cachaça	2000	PMID:25665008
JB848	NOTT12;UFMGR428;CBS10475	Brazil (Aracaju)	frozen pulp of Eugenia uniflora ("pitanga", Myrtaceae)	1999	PMID:25665008
JB852	NOTT16;UFMGA529;CBS10462	Brazil (Belo Horizonte)	must of Brazilian cachaça	1996	PMID:25665008
JB853	NOTT17;UFMGA1000;CBS10465	Brazil (Belo Horizonte)	must of Brazilian cachaça	1996	PMID:25665008
JB854	NOTT18;UFMGR427;CBS10474	Brazil (Aracaju)	frozen pulp of Eugenia uniflora ("pitanga", Myrtaceae)	1999	PMID:25665008
JB858	NOTT22;UFMGA738;CBS10464	Brazil (Belo Horizonte)	must of Brazilian cachaça	1996	PMID:25665008
JB862	NOTT26;NCYC380;CBS10392	ND	raw cane sugar	1953	PMID:25665008
JB864	NOTT28*;ATCC24751;CBS10391;NCYC132	Africa	African Millet Beer	1921	PMID:25665008
JB870	NOTT34;Y0037;CBS10499	South Africa	wine	ND	PMID:25665008
JB872	NOTT36;CBS2775;IFO0347;NBRC0347;NCYC3418	Japan	fermenting molasses	1957	PMID:25665008
JB873	NOTT37;CBS5680;DBVPG6448;NCYC3422	Poland	apple	1965	PMID:25665008
JB874	NOTT38;CBS5682;DBVPG6376;NCYC3421	South Africa	millet beer	1965	PMID:25665008
JB875	NOTT39;CBS7335	Spain	alpechín (water which oozes from a heap of olives)	1988	PMID:25665008
JB878	NOTT42;DBVPG2804	Malta	wine	1963	PMID:25665008
JB879	NOTT43;DBVPG2805	Malta	wine	1963	PMID:25665008
JB884	NOTT48;DBVPG2810	Malta	wine	1963	PMID:25665008
JB899	NOTT63;Y470?	ND	ND	ND	PMID:25665008
JB900	NOTT64;Y831	South Africa	industrial glucose	ND	PMID:25665008
JB902	NOTT66;CBS374	Netherlands (Delft)	molasses	1928	PMID:25665008
JB910	NOTT74;DBVPG6449;CBS1062;IFO0344;NRRLY-1358	ND	cane sugar molasses	ND	PMID:25665008
JB913	NOTT77;CBS1058	Indonesia	molasses	1949	PMID:25665008
JB914	NOTT78;CBS357;DBVPG6280;CLIB834	Jamaica	molasses	1912	PMID:25665008
JB916	NOTT80;CBS352;DBVPG6373	Indonesia	arrack factory (distilled alcoholic drink made from sap of the coconut flower)	1923	PMID:25665008
JB917	NOTT81;CBS1057;DBVPG6375	Sweden	brewer's yeast	1933	PMID:25665008
JB918	NOTT82;CBS1059	Mauritius	cane sugar	1949	PMID:25665008
JB929	NOTT93;CBS1063;DBVPG6450;NRRLY-1362	ND	cane sugar molasses	1934	PMID:25665008
JB930	NOTT94;FRR2208	Australia (Nundah)	glace syrup	ND	PMID:25665008
JB931	NOTT95;FRR2535	ND	raspberry cordial concentrate	1983	PMID:25665008
JB934	CLIB837	France	winemaking	ND	PMID:25665008
JB938	CLIB841	France	winemaking	ND	PMID:25665008
JB939	CLIB842	Spain	winemaking	ND	PMID:25665008
JB942	CLIB845	France	winemaking	ND	PMID:25665008
JB943	CLIB846	France	winemaking	ND	PMID:25665008
JB953	PHAFF65-116	Australia	exudate of a Eucalyptus? Tree	ND	PMID:25665008
JB1110	YHL266;Zimmer1987_0209	ND	red current jelly	1977	PMID:25665008
JB1117	YHL281;Zimmer1987_0208	Holland	malt	1975	PMID:25665008
JB1154	AWRI1875	Australia (Barossa Valley)	ND	ND	PMID:25665008
JB1171	CECT12622;NOTT123;IFI356	ND	grape juice	1982	PMID:25665008
JB1174	CECT12918;NOTT126;IFI2139	ND	grape juice	1982	PMID:25665008
JB1180	Kambucha_YFS276;NOTT132	ND	ND	ND	PMID:25665008
JB1197	NBRC0340;NOTT159;AJ14275;IFO0340;JCM1846	ND	ND	1983	PMID:25665008
JB1205	NBRC10568;NOTT167	ND	ND	ND	PMID:25665008
JB1206	NBRC10569;NOTT168	ND	ND	ND	PMID:25665008
JB1207	NBRC10570;NOTT169	ND	ND	ND	PMID:25665008
JB1468	GZLJ3.34	Guizhou Laojiao Distillery, Changshun, Guizhou		Dec. 2012	NA
JB1508	Santiago Benito V1; HE963293	Spain	Organic Honey	2012	PMID:24929740
JB1469	GZLJ3.36	Guizhou Laojiao Distillery, Changshun, Guizhou		Dec. 2012	NA
JB1470	GZLJ3.41	Guizhou Laojiao Distillery, Changshun, Guizhou		Dec. 2012	NA
JB1471	GZLJ5.6	Guizhou Laojiao Distillery, Changshun, Guizhou		Dec. 2012	NA
JB1472	GZLJ5.28	Guizhou Laojiao Distillery, Changshun, Guizhou		Dec. 2012	NA
JB1473	QT11.6	Gucheng Laojiao Distillery, Qitai, Xinjiang		Sept. 2013	NA
JB1474	XEBLK30.8	Xiaoerbulake Distillery, Xinyuan, Xinjiang		Sept. 2013	NA
JB1475	XEBLK1.1	Xiaoerbulake Distillery, Xinyuan, Xinjiang		Sept. 2013	NA
JB1476	XEBLK2.1	Xiaoerbulake Distillery, Xinyuan, Xinjiang		Sept. 2013	NA
JB1477	XEBLK3.1	Xiaoerbulake Distillery, Xinyuan, Xinjiang		Sept. 2013	NA
JB1478	XEBLK4.1	Xiaoerbulake Distillery, Xinyuan, Xinjiang		Sept. 2013	NA
JB1479	XEBLK7.1	Xiaoerbulake Distillery, Xinyuan, Xinjiang		Sept. 2013	NA
JB1480	XEBLK22.2	Xiaoerbulake Distillery, Xinyuan, Xinjiang		Sept. 2013	NA
JB1481	XEBLK27.3	Xiaoerbulake Distillery, Xinyuan, Xinjiang		Sept. 2013	NA
JB1482	XEBLK29.10	Xiaoerbulake Distillery, Xinyuan, Xinjiang		Sept. 2013	NA
JB1483	XEBLK30.2	Xiaoerbulake Distillery, Xinyuan, Xinjiang		Sept. 2013	NA
JB1484	XEBLK31.1	Xiaoerbulake Distillery, Xinyuan, Xinjiang		Sept. 2013	NA
-	CECT1375;IFI935	Spain		1983	
-	CECT1376; IFI936	Spain		1983	
-	CECT12512; IFI87	Spain	must	1999	
-	CECT12513; IFI88	Spain	must	1999	

### First phase of *S*. *pombe* strain selection

Starter cultures were grown from 100 μL of each yeast suspension, cultivated in 5 mL volumes of YEPD at 25°C for 24 h. This procedure was performed in triplicate before the final inoculation of 1 mL in the fermentative medium. 1 mL (10^8^ CFU/mL) of these starters cultures were then inoculated into tubes containing 9 ml of sterilised concentrated must (Dream Fruits S.A., Quero, Toledo, Spain), which was diluted to 212 g/L glucose + fructose and enriched with 4 g/L malic acid (Panreac, Barcelona, Spain) (final pH 3.1) to simulate acidic musts where the use of *S*. *pombe* is more recommended in order to increase wine quality [3,5]. After 21 days of fermentation, enzymatic analysis was performed for glucose + fructose, malic acid and acetic acid (S1 Table). This experiment was performed in triplicate for each studied strain.

### Yeasts used in the second phase of *S*. *pombe* strain selection

The yeast strains used in the second selection phase included *S*. *pombe* IFI936/CECT12774 and IFI935/CECT1376 and *S*. *cerevisiae* IFI87/CECT12512 and IFI88/CECT12513 from the Spanish Type Culture collection. The *S*. *pombe* strains JB899/Y470, JB873/NCYC3422 and JB917/CBS1057 were selected during the first phase of the study. The strain V1 was selected in a previous study [5].

### Second phase of *S*. *pombe* strain selection

Microfermentations were performed using 200 ml aliquots of concentrated must (Dream Fruits S.A., Quero, Toledo, Spain), which were diluted to glucose + fructose (G + F) concentrations of 211 g/L, enriched to 4.3 g/L malic acid (Panreac, Barcelona, Spain) (final pH 3.08), with 60 g/L of Actimax Natura (Agrovín S.A., Ciudad Real, Spain) added to provide nutrition. Each microfermentator was inoculated with 1 mL of liquid YEPD medium containing 10^7^ CFU/mL (determined using a Thomas chamber) of one of the above-mentioned yeasts. All fermentations were performed in 250-ml flasks, which were sealed with a Muller valve and filled with 98% H_2_SO_4_ (Panreac, Barcelona, Spain) to allow the release of CO_2_ and to prevent microbial contamination [31]. The temperature was maintained at 25°C. The fermentations proceeded without aeration, oxygen injection or agitation. All fermentations were performed in triplicate.

The glucose + fructose, malic acid, acetic acid and pyruvic acid contents of the fermentations were monitored over a period of 14 days. The pH, urea, citric acid, glycerol, alcohol content, volatile compounds, amino acids and biogenic amine concentrations were determined at the end of the fermentation.

### Measurements of biochemical compounds and pH

Glucose + fructose, malic acid, L-lactic acid, acetic acid, pyruvic acid, urea, glycerol and pyruvic acid analyses (Table 2) were conducted using a Y15 Autoanalyser (Biosystems, Barcelona, Spain). The kits to perform the analyses were obtained from Biosystems [32]. Alcohol content was determined by using the boiling method GAB Microebu [33]. The pH was measured with a Crison pH Meter Basic 20 (Crison, Barcelona, Spain).

**Table 2:** Final analysis of fermentations performed by the studied strains. Selected strains are JB899/Y470, JB917/CBS1057, JB873/NCYC3422 and V1, non-selected *S*. *pombe* strains from the Spanish culture collection (IFI936/CECT12774 and IFI935/CECT1376) and selected *S*. *cerevisiae* strains (IFI87/CECT12512 and IFI88/CECT12513).

Compounds	899	917	873	V1	935	936	87	88
l-Lactic Acid (g/L)	0.00 ± 0.00a	0.00 ± 0.00a	0.00 ± 0.00a	0.00 ± 0.00a	0.00 ± 0.00a	0.00 ± 0.00a	0.00 ± 0.00a	0.00 ± 0.00a
l-Malic Acid (g/L)	0.02 ± 0.01a	0.01 ± 0.01a	0.02 ± 0.01a	0.01 ± 0.01a	1.02 ± 0.11b	0.01 ± 0.01a	3.16 ± 0.14c	3.72 ± 0.08d
Acetic Acid (g/L)	0.24 ± 0.02a	0.29 ± 0.02b	0.33 ± 0.03b	0.30 ± 0.02b	0.93 ± 0.05c	0.98 ± 0.07c	0.21 ± 0.02a	0.24 ± 0.02a
Residual Sugar (g/L)	1.77 ± 0.52a	1.89 ± 0.64a	2.02 ± 0.58a	2.12 ± 0.36a	2.11 ± 0.43a	1.99 ± 0.35a	1.74 ± 0.26a	2.22 ± 0.31a
Glycerol (g/L)	8.32 ± 0.16a	8.88 ± 0.21c	8.02 ± 0.09a	8.91 ± 0.18c	8.14 ± 0.13a	8.48 ± 0.11b	8.36 ± 0.14ab	8.14 ± 0.08a
pH	3.44 ± 0.02d	3.45 ± 0.02d	3.44 ± 0.02d	3.42 ± 0.02d	3.36 ± 0.03c	3.45 ± 0.03d	3.14 ± 0.02 b	3.11 ± 0.02a
Urea (mg/L)	0.42 ± 0.02a	0.48 ± 0.02b	0.39 ± 0.03a	0.44 ± 0.02ab	0.42 ± 0.03a	0.51 ± 0.03b	2.48 ± 0.03c	2.52 ± 0.04c
Citric Acid (g/L)	0.27 ± 0.01a	0.29 ± 0.02a	0.29 ± 0.03a	0.28 ± 0.02a	0.28 ± 0.03a	0.27 ± 0.02a	0.29 ± 0.02a	0.28 ± 0.01a
Alcohol (% *v/v*)	12.15 ± 0.02b	12.04 ± 0.02a	12.26 ± 0.03c	12.02 ± 0.02a	12.24 ± 0.02c	12.13 ± 0.03b	12.43 ± 0.02d	12.44 ± 0.03d

Results are the mean ± SD of three replicates. Means in the same row with the same letter are not significantly different (*p* < 0.05).

### Quantification of volatile compounds

Volatile compounds (Table 3) were quantified by headspace gas chromatography–mass spectrometry (HS-GC-MS). Analyses were carried out using a Perkin-Elmer Clarus 500 gas chromatograph with a flame ionization detector, coupled to a mass spectrometer single quadrupole Clarus 560 S, all coupled to an automatic headspace sampler Turbomatrix 110 Trap (Perkin-Elmer, Massachusetts, USA). The headspace sampler conditions were: temperature of thermostating: 80°C, time of thermostating: 45 min, type of trap: Tenax TA, cycles of purge and trap: 4, temperature of trap capture: 45°C, desorption temperature of the trap: 290°C, time of dry trap purge: 10 min, desorption time of trap: 2 min, trap cleaning time: 5 min, needle temperature: 110°C, temperature of HS-GC transfer line: 150°C, vial pressure: 30 psi and constant pressure column: 28 psi. A FFAP capillary column (60 m × 0.25 mm DI x 0.25 μm film thickness) was used. Helium (Air Liquide, España) was used as carrier gas. Gradient analysis was run using the following temperature program: 40°C (3 min); 40–80°C (2°C/min); 80–180°C (3°C/min); and 210°C (5 min). Identification of individual compounds was based on a comparison of the obtained mass spectra of the individual chromatographic peaks with those valid for the standards and available from the National Institute of Standards and Technology (Gaithersburg, MD) software library. We also compared the retention times valid for individual peaks from the wine samples with those of the known volatile components use as standard patterns. To this effect, we used Gas chromatography quality compounds as the sets of the volatile standards (Fluka, Sigma–Aldrich Corp., Buchs SG, Switzerland).

**Table 3:** Final analysis of volatile compounds performed by the studied strains during fermentations. Selected *S*. *pombe* strains (JB899/Y470, JB917/CBS1057, JB873/NCYC3422 and V1), non-selected *S*. *pombe* strains from the Spanish culture collection (936/CECT12774 and IFI 935/CECT1376) and selected *S*. *cerevisiae* strains (IFI 87/CECT12512 and IFI 88/CECT12513) from the Spanish culture collection.

Compounds (mg/L)	899	917	873	V1	935	936	87	88
Acetaldehyde	16.46±1.52a	17.82±2.31a	16.99±1.78a	18.13±2.42a	32.46±3.11b	36.52±2.97b	16.18±1.86a	17.56±2.06a
Ethyl lactate	6.32±0.43a	6.58±0.47a	6.52±0.49a	6.39±0.45a	7.76±0.58b	7.82±0.54b	7.23±0.48ab	7.54±0.38b
Ethyl acetate	16.32±1.87a	18.11±2.16a	18.33±2.28a	18.62±2.39a	82.41±4.54b	89.13±5.26b	17.45±1.54a	19.23±3.06a
Diacetyl	2.24±0.16a	2.67±0.32a	2.48±0.26a	2.32±0.24a	2.91±0.55a	2.84±0.48a	2.29±0.33a	2.36±0.38a
Isoamyl acetate	2.28±0.28a	2.21±0.32a	2.44±0.43a	2.33±0.28a	2.68±0.42a	2.59±0.38a	3.77±0.51b	3.62±0.48b
2- Phenyl ethyl acetate	5.25±0.21a	5.36±0.28a	5.29±0.25a	5.38±0.19a	5.48±0.25a	5.54±0.27a	6.41±0.28b	6.34±0.26b
1-Propanol [mg/L]	11.42±1.52a	10.12±1.36a	13.26±2.08ab	10.73±1.84a	18.99±3.62b	19.76±3.93b	28.45±4.01c	31.33±4.13c
Isobutanol	9.31±1.46a	8.57±1.88a	9.86±1.74a	8.94±2.02a	12.11±2.36a	13.52±2.58ab	17.51±2.24b	18.13±2.48b
1-Butanol	5.13±1.03a	5.45±1.09a	5.36±1.14a	5.28±1.19a	7.23±1.26ab	7.44±1.42ab	7.81±1.12b	7.96±1.23b
3-Methyl-butanol	11.36±1.56a	12.16±1.36a	11.98±1.43a	12.28±1.86a	18.44±1.68b	18.88±1.95b	26.12±2.33c	28.06±2.54c
2-Methyl-butanol	23.34±2.36a	24.48±2.43a	25.68±2.67a	24.19±1.88a	31.08±2.09b	33.16±2.24b	40.44±3.16c	42.83±4.23c
Isobutyl acetate	n.d.	n.d.	n.d.	n.d.	n.d.	n.d.	n.d.	n.d.
Ethyl butyrate	n.d.	n.d.	n.d.	n.d.	n.d.	n.d.	n.d.	n.d.
2-Phenyl-ethanol	21.76±2.11a	22.28±1.98a	21.44±1.78a	22.35±2.23a	24.17±1.79a	24.66±1.68a	28.97±1.82b	29.12±1.96b
Hexanol	1.31±0.12a	1.39±0.14a	1.36±0.15a	1.34±0.16a	1.42±0.14a	1.46±0.16a	2.45±0.21b	2.48±0.26b

Results are the mean ± SD of three replicates. Means in the same row with the same letter are not significantly different (*p* < 0.05), n.d. = not detected.

### Quantification of biogenic amines

The studied biogenic amines (Table 4) were analysed using a Jasco (Tokyo, Japan) UHPLC chromatograph series X-LCTM, equipped with a Fluorescence detector 3120-FP. Gradients of solvent A (methanol/acetonitrile, 50:50, v/v) and B (sodium acetate /tetrahydrofuran, 99:1, v/v) were used in a C18 (HALO, USA) column (100 mm × 2.1 mm; particle size 2.7 μm) as follows: 60% B (0.25 ml/min) from 0 to 5 min, 60–50% B linear (0.25 ml/min) from 5 to 8 min, 50% B from 8 to 9 min, 50–20% B linear (0.2 ml/min) from 9 to 12 min, 20% B (0.2 ml/min) from 12 to 13 min, 20–60% B linear (0.2 ml/min) from 13 to 14.5 min, and re-equilibration of the column from 14.5 to 17 min. Detection was performed by scanning in the 340–420 nm range. Quantification was performed by comparison against external standards of the studied amines. The different amines were identified by their retention times.

**Table 4:** Final analysis of biogenic amines. Fermentations by selected *S*. *pombe* strains (JB899/Y470, JB917/CBS1057, JB873/NCYC3422 and V1), non-selected *S*. *pombe* strains from the Spanish culture collection (936/CECT12774 and IFI 935/CECT1376) and selected *S*. *cerevisiae* strains (IFI 87/CECT12512 and IFI 88/CECT12513).

Compounds	899	917	873	V1	935	936	87	88
Histamine (mg/L)	0.32 ± 0.03a	0.36 ± 0.04a	0.32 ± 0.04a	0.34 ± 0.03a	0.38 ± 0.04a	0.36 ± 0.02a	0.33 ± 0.02a	0.32 ± 0.03a
Tiramine (mg/L)	0.16 ± 0.01a	0.18 ± 0.04a	0.16 ± 0.02a	0.17 ± 0.06a	0.18 ± 0.04a	0.16 ± 0.02a	0.16 ± 0.02a	0.15 ± 0.02a
Phenylethylamine (g/L)	n.d.	n.d.	n.d.	n.d.	n.d.	n.d.	n.d.	n.d.
Putrescine (g/L)	0.43 ± 0.03a	0.46 ± 0.06a	0.42 ± 0.05a	0.44 ± 0.04a	0.43 ± 0.05a	0.45 ± 0.07a	0.42 ± 0.03a	0.41 ± 0.04a
Cadaverine (g/L)	0.56 ± 0.03ab	0.55 ± 0.05ab	0.49 ± 0.05a	0.52 ± 0.04a	0.52± 0.03a	0.53 ± 0.03a	0.63 ± 0.03b	0.62 ± 0.04b

Results represent the mean ± SD for three replicates. Means in the same row with the same letter are not significantly different (*p* < 0.05), n.d. = not detected.

### Analytical determinations of amino acids

Selected amino acids (Table 5) were analysed using a Jasco (Tokyo, Japan) UHPLC chromatograph series X-LCTM, equipped with a Fluorescence detector 3120-FP. Gradients of solvent A (methanol/acetonitrile, 50:50, v/v) and B (sodium acetate /tetrahydrofuran, 99:1, v/v) were used in a C18 (HALO, USA) column (100 mm × 2.1 mm; particle size 2.7 μm) as follows: 90% B (0.25 mL/min) from 0 to 6 min, 90–78% B linear (0.2 mL/min) from 6 to 7.5 min, 78% B from 7.5 to 8 min, 78–74% B linear (0.2 mL/min) from 8 to 8.5 min, 74% B (0.2 mL/min) from 8.5 to 11 min, 74–50% B linear (0.2 mL/min) from 11 to 15 min, 50% B (0.2 mL/min) from 15 to 17 min, 50–20% B linear (0.2 mL/min) from 17 to 21 min, 20–90% B linear (0.2 mL/min) from 21 to 25 min, and re-equilibration of the column from 25 to 26 min. Detection was performed by scanning in the 340–455 nm range. Quantification was performed by comparison against external standards of the studied amino acids. The different amino acids were identified by their retention times.

**Table 5:** Final analysis of amino acids. Fermentations by selected *S*. *pombe* strains (JB899/Y470, JB917/CBS1057, JB873/NCYC3422 and V1), non-selected *S*. *pombe* strains from the Spanish culture collection (936/CECT12774 and IFI 935/CECT1376) and selected *S*. *cerevisiae* strains (IFI 87/CECT12512 and IFI 88/CECT12513) from the Spanish culture collection.

Compounds (mg/L)	899	917	873	V1	935	936	87	88
Aspartic acid	10.82±1.51b	11.96±1.68b	11.42±1.33b	12.16±1.78b	21.52±2.46c	18.83±2.13c	7.36±0.68a	8.12±0.74a
Asparagine	19.68±1.56b	20.25±1.72b	19.93±1.84b	21.37±2.12b	30.18±2.89c	29.06±2.52c	16.34±1.09a	17.45±1.14a
Serine	6.25±0.89b	6.88±1.02b	6.47±0.78b	7.43±0.93b	11.69±1.36c	10.78±1.27c	3.18±0.35a	3.64±0.48a
Histidine	81.32±3.59a	83.29±3.52a	82.94±2.89a	84.32±3.46a	92.35±4.48c	94.88±4.69c	65.47±3.18a	69.34±3.46a
Glycine	28.42±1.33a	27.83±1.16a	28.48±1.26a	29.68±1.44a	39.68±1.77b	41.12±2.24b	27.43±1.11a	28.56±1.23a
Arginine	66.52±4.62b	64.52±3.96b	63.14±3.54b	69.52±5.43b	78.21±6.22c	76.52±5.84c	42.43±3.53a	40.88±3.17a
Threonine	53.17±3.46c	51.19±2.98c	46.23±2.72c	49.94±2.56c	37.46±2.97b	36.82±2.64b	24.72±1.82a	22.58±2.01a
Alanine	27.31±2.07a	28.43±2.16a	32.05±3.08a	29.86±3.17a	42.57±3.21b	43.26±3.45b	31.68±2.49a	29.72±2.28a
Tyrosine	9.82±0.68b	10.13±0.62b	11.28±0.86bc	10.22±0.75b	12.88±0.93c	13.31±1.14c	4.48±0.26a	4.69±0.34a
Valine	4.73±0.31c	5.29±0.44c	4.82±0.35c	5.18±0.42c	2.96±0.27b	3.18±0.31b	1.25±0.19a	1.14±0.16a
Tryptophan	1.41±0.26b	1.46±0.22b	1.59±0.28b	1.54±0.23b	1.76±0.23b	1.81±0.29b	0.52±0.13a	0.46±0.07a
Phenylalanine	5.54±0.42b	5.26±0.38b	5.69±0.51b	5.89±0.54b	7.62±0.86b	7.98±1.02 b	3.65±0.29a	3.78±0.33a
Isoleucine	12.04±0.74c	12.56±0.78c	12.64±0.76c	13.24±0.88c	8.17±0.63b	8.35±0.68b	2.45±0.32a	2.62±0.36a
Leucine	15.18±0.93c	14.69±0.87c	15.92±1.05c	15.86±0.92c	10.22±0.81b	11.46±0.98b	3.87±0.68a	3.98±0.673a
Ornithine	18.36±1.65a	17.87±1.42a	18.45±1.72a	19.88±1.78a	19.93±1.86a	20.98±1.89a	29.48±2.27b	30.74±2.36b
Lysine	12.27±0.96b	12.59±0.88b	12.72±0.91b	14.13±1.02b	23.56±1.94c	24.56±2.23c	6.16±0.85a	6.77±0.94a
Methionine	2.68±0.43b	2.89±0.36b	2.73±0.41b	3.27±0.52b	6.26±0.89c	6.47±0.97c	1.23±0.19a	1.29±0.22a

Results represent the mean ± SD for three replicates. Means in the same row with the same letter are not significantly different (*p* < 0.05).

### Fermentation in stress conditions

Study of fermentation in stress caused by ethanol, sulphur dioxide and the osmotic stress caused by salts (NaCl and KCl), were performed based on methods described previously for *S*.*cerevisiae* [34]. 15 ml sealed test tubes contatining 10 ml of sterilized grape must were inoculated with the studied strains. The grape must final sugar concentration was 206 g/L diluted from a concentrated grape must (Dream Fruits S.A., Quero, Toledo, Spain) that was corrected to the respective stressful conditions before yeast inoculation: Ethanol (6%; 10%; 12% and 14%), KCl (0.75 M), NaCl (1.5 M) (both osmotic stress) and KHSO_3_ (150 and 300 mg/L) (sulphur dioxide). Ethanol, KCl, NaCl products were from Panreac (Panreac, Barcelona, Spain) and KHSO_3_ was from Agrovín S.A. (Alcázar de San Juan, Spain). The testing tubes were inoculated to a cellular density of 5x10^6^ cells/mL with the studied strains: *S*. *pombe* strains (JB899/Y470, JB917/CBS1057, JB873/NCYC3422, JB4/ NCYC683, JB837/ NOTT1 and V1), non-selected *S*. *pombe* strains from the Spanish culture collection (936/CECT12774 and IFI 935/CECT1376) and selected *S*. *cerevisiae* strains (IFI 87/CECT12512 and IFI 88/CECT12513) from the Spanish culture collection. Final optical density was determined at 640 nm after 24 h of incubation at 25°C by an Y15 spectrophotometer (Biosystems, Barcelona, Spain). The maximal ethanol production was estimated through the fermenting power method [35,36]. In this case the original concentrated grape must (Dream Fruits S.A., Quero, Toledo, Spain) was diluted to a sugar concentration of 300 g/L, the sealed flasks with Müller trapflasks (Alamo, Madrid, Spain) were incubated at 25°C. All the tests were performed in triplicate.

### Sensory analysis

For a sensory analysis, fermentations triplicate with *S*. *pombe* strains (JB899/Y470, JB873/NCYC3422, JB917/CBS1057, JB4/NCYC683, JB837/ NOTT1, 936/CECT12774, IFI935/CECT1376 and V1) and *S*. *cerevisiae* strains (IFI 87/CECT12512 and IFI88/CECT12513) were performed with microvinifications with similar methodology to previously described [13, 15]. Sealed 1 L flasks with a Müller trapflasks (Alamo, Madrid, Spain) containing 800 ml of sterilised (115°, 15 min) concentrated must (Dream Fruits S.A., Quero, Toledo, Spain), which was diluted to 202 g/L glucose + fructose and enriched with 4 g/L malic acid (Panreac, Barcelona, Spain) (final pH 3.11). The flasks were inoculated with the studied strains to initial population of 10^6^ CFU/mL. The completion of the fermentation process was verified by weight loss and confirmation that final enzymatic analysis of glucose + fructose was below 3 g/L after fermenting at 25°C. After fermentation, wines were racked and stored for 7 days at 4°C in 750 mL wine bottles. The final product was bottled in 350 mL wine bottles, sealed bottles and stored horizontally in a climate chamber at 4°C for two weeks until sensory evaluation.

Final wines were assessed (in a blind test) by a panel of 10 experienced wine tasters, all members of the staff of the Food Technology Department of the Technical University of Madrid, Spain. Assessments took place in standard sensory analysis chambers with separate booths. Following the generation of a consistent terminology by consensus, one visual descriptor, four aromas and four taste attributes were chosen to describe the wines. Formal assessment consisted of two sessions held on different days where wine tasters tasted all fermented triplicates. The panellists used a 10-cm unstructured scale, from 0 (no character) to 10 (very strong character), to rate the intensity of the 10 attributes.

### Statistical analyses

All statistical analyses were performed using PC Statgraphics v. 5 software (Graphics Software Systems, Rockville, MD, USA). The significance was set to p <0.05 for the ANOVA matrix F value. The multiple range test was used to compare the means.

## Results and Discussion

### First phase of *S*. *pombe* strain selection

**Fig 1:**
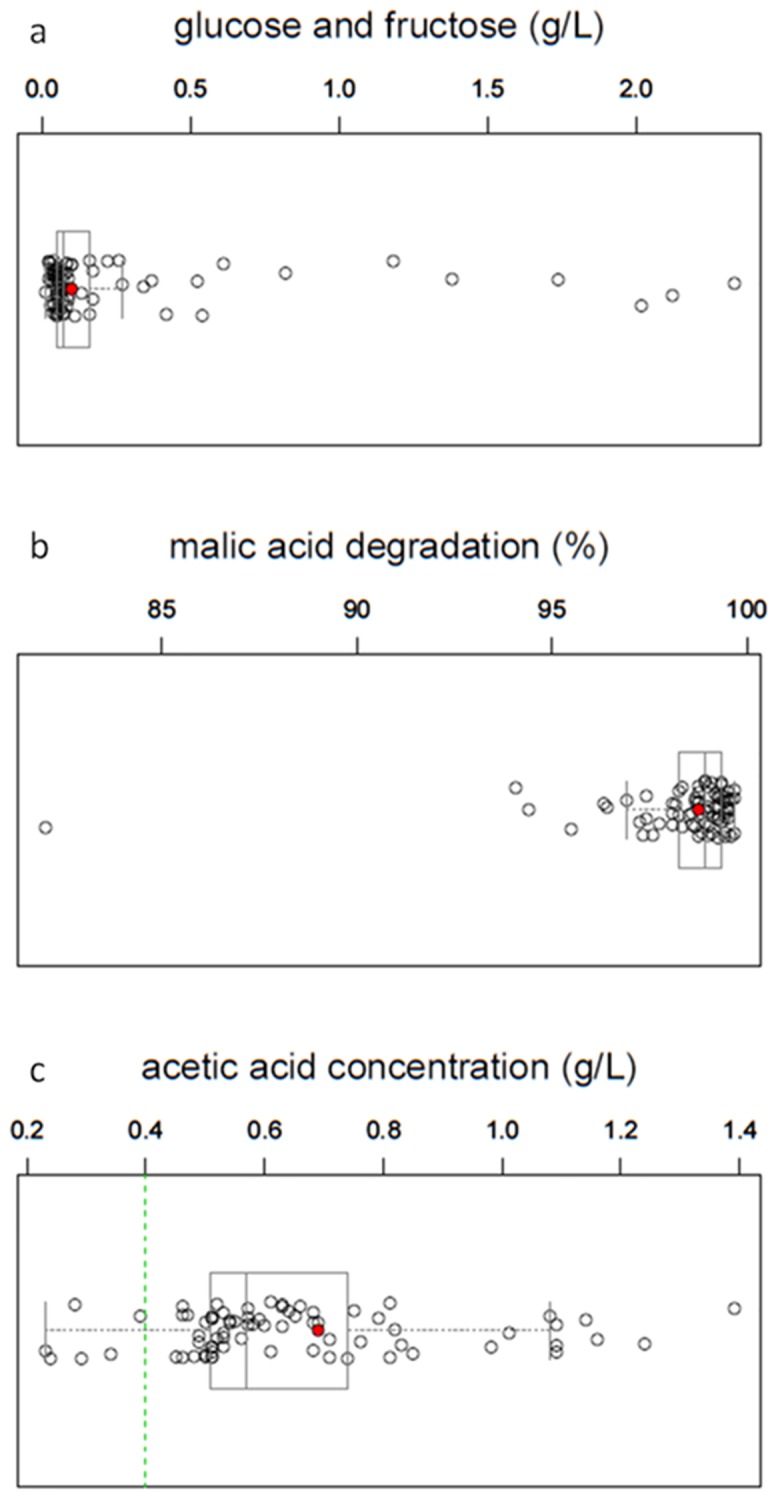
(a) Glucose + fructose concentrations (g/L); (b) malic acid degradation (%); (c) acetic acid concentration (g/L). Scatterplots and box-and-whisker plots for the mean values of the final fermentations for the 75 studied *S*. *pombe* strains after 21. The red points indicate the values of the standard laboratory reference strain (Leupolds, 972, 975, 978), green line indicates values of strains with low levels of acetic acid (<0.4 g/L).

A specific advantage of *Schizosaccharomyces* yeast in winemaking is that it degrades malic acid. To examine this with our strains, we initiated fermentation of grape must supplemented with malic acid (4 g/L). After fermenting the 75 *S*. *pombe* strains in test tubes for 21 days, we measured malic acid. We also measured two basic parameters, residual glucose + fructose and acetic acid production, which are typically used in the selection of fermentative yeasts of the genus *Saccharomyces*, the main yeast genus used in winemaking. The final concentrations of glucose + fructose after fermentation were close to 0 g/L (Fig 1a). This finding is in agreement with data reported by other authors, and is indicative of strong fermentative power [37,3]. This property allows the production of dry wines that are most popular in the world market.

The degradation of malic acid, which currently represents the primary industrial application of *Schizosaccharomyces* yeasts, was nearly 100% in all the studied strains (Fig 1b). These data are comparable to those from other authors, who reported malic acid degradation rates ranging between 75 and 100% [38].

Out of the 75 initial *S*. *pombe* strains tested, however, only five strains (JB4/NCYC683, JB837/UWOPS92.229.4, JB873/NCYC3422, JB899/Y470 and JB917/CBS1057) showed moderate final concentrations of acetic acid (<0.4 g/L) (Fig 1c). According to these results, just 6.5% of the *S*. *pombe* strains could be used for producing quality wine. In a previous study, a similarly low rate of 5% was reported [5]. Three of these strains (JB837, JB873 and JB899) showed acetic acid concentration lower than 0.3 g/L. These data demonstrate that it is possible to identify *S*. *pombe* strains that can produce quality wines (Fig 1c).

### Comparison between pre-selected *S*. *pombe* strains and other culture collection strains

After identifying the three *S*. *pombe* strains that show low acetic acid production, we studied additional features in detail. Microvinifications (200 mL) were performed to verify the acetic acid data and to confirm the proper fermentative ability of the pre-selected strains. In this portion of the study, the pre-selected *S*. *pombe* strains (JB837, JB873 and JB899) were compared with two *S*. *cerevisiae* strains (87/CECT12512 and 88/CECT12513) and with three other *S*. *pombe* strains (V1, 935/CECT1376 and 936/CECT12774) that had been tested previously [5].

#### Acetic acid

**Fig 2:**
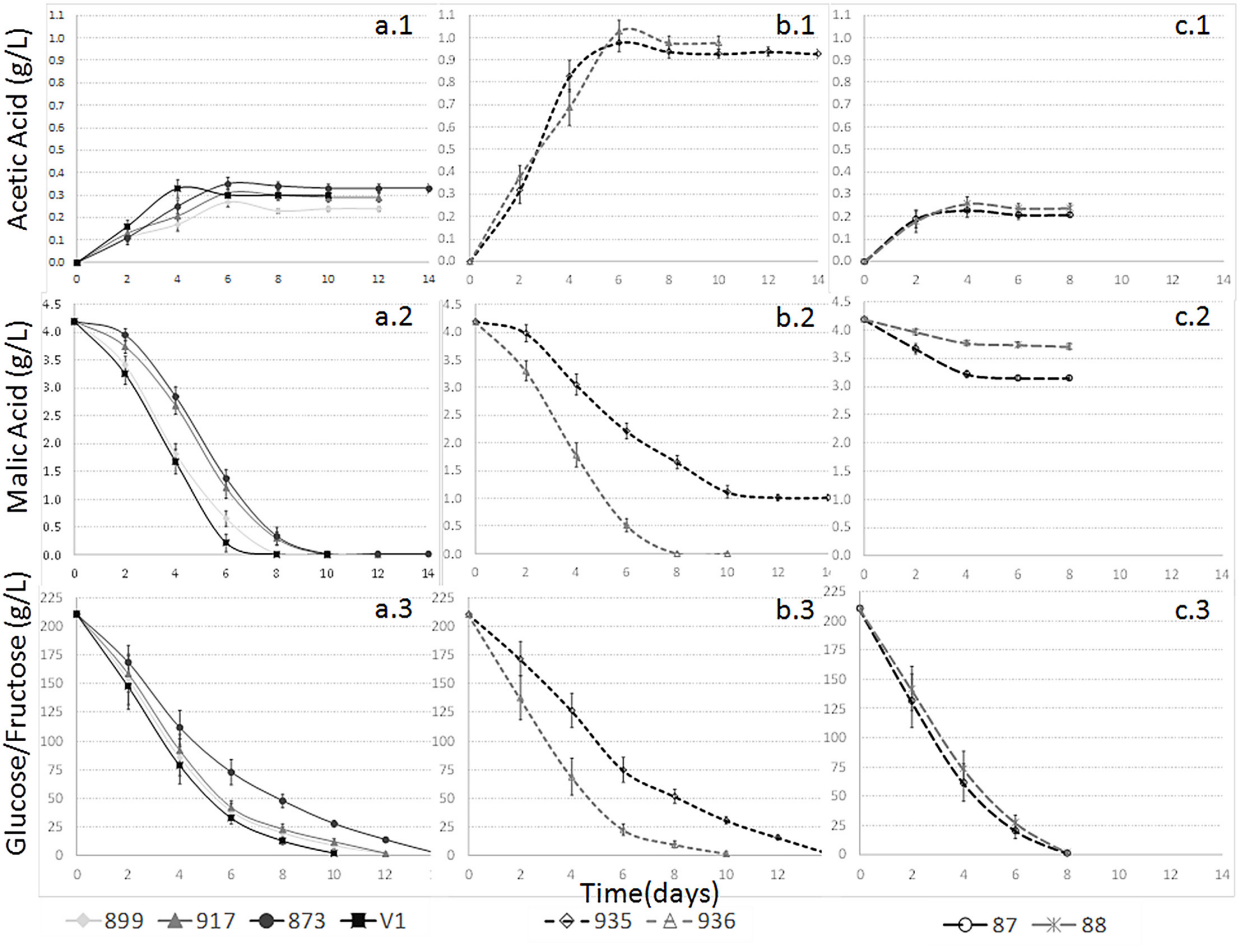
Fermentation kinetics of acetic acid (1), malic acid (2) and glucose + fructose (3) for (a) selected *S*. *pombe* strains (JB899/Y470, JB917/CBS1057, JB873/NCYC3422 and V1); (b) non-selected *S*. *pombe* strains from the collection (935 and 936); (c) selected *S*. *cerevisiae* strains (87 and 88).

The maximal final concentrations of acetic acid were approximately 0.3 g/L (Fig 2.a.1; Table 2) for all selected strains, while the *S*. *pombe* strains from the Spanish Type Culture Collection produced acetic acid concentrations of up to 1 g/L (Fig 2.b.1; Table 2). Therefore, when used as a single strain during fermentation, the latter strains are not suitable for the production of quality wines [14,5].

#### Malic acid

The malic acid degradation was almost complete for most of the studied *S*. *pombe* strains (Fig 2.a.2 and 2.b.2; Table 2), although the degradation kinetics differed. The two *S*. *cerevisiae* strains degraded 11–24% of the initial malic acid content in the must (Fig 2.c.2; Table 2). Several authors have proposed similarly high malic acid degradation for yeast belonging to other genera than *Schizosaccharomyces* [5, 39–43]. The malic acid reduction influenced the final pH of the wine (Table 2), as *S*. *pombe* fermentations showed up to 0.34 higher pH values than *S*. *cerevisiae* fermentations.

#### Sugar consumption kinetics

The consumption kinetics of glucose + fructose was more rapid in the *S*. *cerevisiae* strains than in most studied *S*. *pombe* strains (Fig 2.a.3, 2.b.3 and 2.c.3; Table 2). Similar results have been described before [6]. Nevertheless, differences in degradation kinetics of glucose + fructose between the studied *S*. *pombe* strains were evident (Fig 2.a.3 and 2.b.3).

#### Pyruvic acid

**Fig 3:**
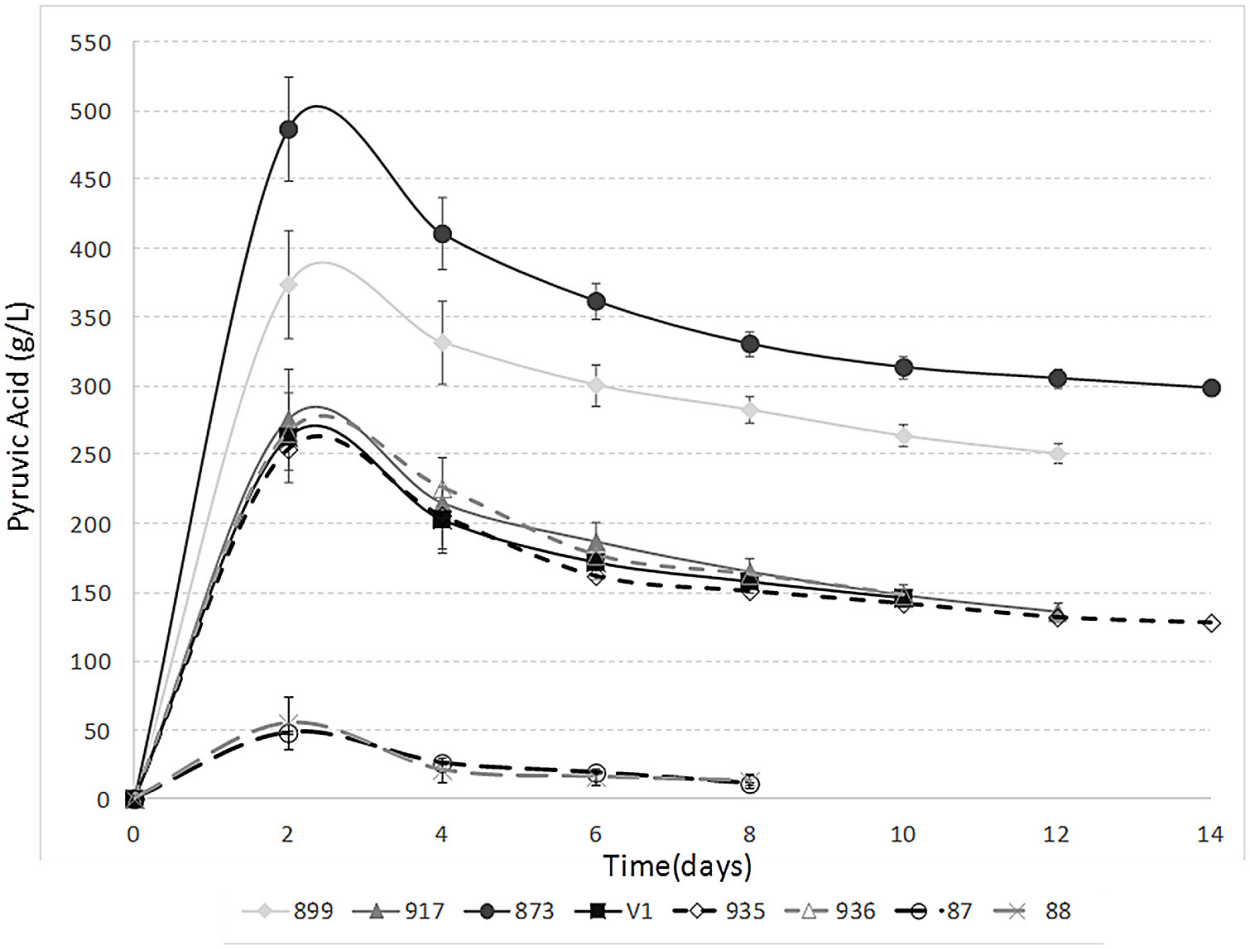
Fermentation kinetics of pyruvic acid for selected *S*. *pombe* strains (JB899/Y470, JB917/CBS1057, JB873/NCYC3422 and V1), non-selected *S*. *pombe* strains from the collection (935 and 936) and selected *S*. *cerevisiae* strains (87 and 88).

All studied *S*. *pombe* strains produced more pyruvic acid than the *S*. *cerevisiae* yeasts (Fig 3). Strain JB873 yielded a maximum pyruvic acid concentration of 487 mg/L after 48 h of fermentation, which is a slightly higher concentration than those obtained in previous studies [5,6]. The other *S*. *pombe* strains provided a maximum pyruvic acid content that varied from 254 to 276 mg/L, except for the case of *S*. *pombe* JB899 that reached a pyruvic acid content of 374 mg/L (Fig 3). Previous pyruvic acid studies with *S*. *cerevisiae* and non-*Saccharomyces* species other than *Schizosaccharomyces* showed maximum values of about 150 mg/L during the first stages of fermentation [43]. These values are substantially lower than those obtained for the studied *S*. *pombe* strains. There is a strong correlation between the amount of pyruvic acid released by yeasts and the formation of vitisin A [3,5,15]; vitisin A is a stable pigment that influences wine colour quality. Thus, the selected strains with high pyruvate production rates such as JB873 and JB899 may be of interest in red wine production.

#### Glycerol

*Schizosaccharomyces* species have previously been reported to produce more glycerol than *Saccharomyces* species [15]. In this study, final levels of glycerol varied from 8.02 g/L to 8.91 g/L (Table 2). Even though two *S*. *pombe* strains showed the highest levels (V1 and JB917), several differences were observed between the studied strains, and not in every case were the values higher than those obtained for the two *S*. *cerevisiae* strains. Increased glycerol content has been described as one of the main contributors of non-*Saccharomyces* strains on wine quality [1,44].

#### Ethanol

Among all strains tested, the final ethanol levels varied from 12.04 to 12.44 (% vol/vol) (Table 2). The fermentations involving *S*. *pombe* produced very similar ethanol levels to the *S*. *cerevisiae* strains we examined (Table 2). Among the *S*. *pombe* strains, only slight differences in final ethanol levels of less than 0.24 (% vol/vol) were evident. In contrast other authors have shown that some non-*Saccharomyces* types of yeast produce lower ethanol yields than *Saccharomyces* [45–47]. The sugar metabolism can also be used to produce compounds other than ethanol, such as glycerol or pyruvic acid, or to increase the yeast biomass [48,49]. Previous studies with *S*. *pombe* showed similar results to those obtained here [13]. Other authors observed lower final ethanol levels using other non-*Saccharomyces* species under specific high aeration conditions [50,51].

#### Urea

The urea content of the finished wines was lower in the fermentations involving *S*. *pombe*, with values less than 0.5 mg/L (Table 2). This result can be attributed to the special ability of the *Schizosaccharomyces* genus to produce urease [12,52]. This enzymatic activity also could reduce the initial level of precursors for ethyl carbamate (one of the most toxic compounds reported in wine) [3,15]. This factor is becoming increasingly important as ethyl carbamate is a known carcinogen that is present in a variety of fermented foods [53].

#### Citric acid and lactic acid

No differences were evident with respect to citric and lactic acid (Table 2), as no wine performed malolactic fermentation [6] or *Lachancea thermotolerans* species were used [54]. Note that after a traditional malolactic fermentation process by lactic bacteria, all citric acid could be converted into acetic acid; this collateral effect increases the final acetic acid content [6] and consequently slightly reduces wine quality.

#### Volatile aromas

Higher alcohols were produced in higher total concentrations by *S*. *cerevisiae* fermentations (Table 3). Some differences were observed between the studied *S*. *pombe* strains (Table 3). Other authors described non-*Saccharomyces* yeasts as lower producers of higher alcohols than *S*. *cerevisiae* [42,43,54–57], and much strain variability has been reported [58,59]. This finding could be of interest in facilitating the making of wines with typicity for specific grape varieties or to increase wine complexity [60]. Similarly, the tested *S*. *pombe* strains produced less esters than the *S*. *cerevisiae* strains. No differences between the *S*. *pombe* and *S*. *cerevisiae* strains were observed with respect to compounds considered negative, such as ethyl acetate and diacetyl. However, those compounds could increase after a malolactic fermentation process performed by lactic bacteria [15]. Significant differences in compounds such as acetaldehyde and ethyl acetate were evident between the different *S*. *pombe* strains.

#### Biogenic amines

During the past years, harmful effects of biogenic amines [61–64] have been demonstrated, which now constitutes a serious matter in food safety that must be taken into account. A histamine value of 2 mg/L is considered the maximum level [65] in some countries. All studied strains reported values below that threshold (Table 4). The study shows that *S*. *pombe* does not produce higher levels of biogenic amines than *S*. *cerevisiae*. Subtle statistical differences were observed in the case of cadaverine (Table 4), but this biogenic amine is not produced by yeasts, and the slight differences could reflect that some strains remove biogenic amines during the fermentation process; similar processes have been described before [66]. However, most biogenic amines are produced during wine ageing and malolactic fermentation [67], as they are compounds produced primarily by lactic acid bacteria [68,69]. Thus, the wines that still contain malic acid (Fermentations by *S*. *cerevisiae*) could increase their biogenic amine contents later on [6,16].

#### Amino acids

*S*. *pombe* fermentations showed higher final levels in most amino acids (Table 5). Similar results were obtained before, as it has been described as demanding less nitrogen [14] and releasing more nitrogen [7] than *S*. *cerevisiae*. However some differences were observed among the studied strains (Table 5). *S*. *cerevisiae* fermentations produced a higher final level in ornithine. The observed differences in threonine, valine, isoleucine and leucine explain the differences measured for higher alcohols (Table 5), because they are precursors of 1-propanol, isobutanol, 2-methylbutanol and 3-methylbutanol, respectively (Table 5) [43]. The statistical differences reported for histidine, tyrosine and lysine show that *S*. *pombe* fermentations increase the content of some biogenic amine precursors [65,67]. Nevertheless, the transformation of some of those precursors to biogenic amines takes place during the malolactic fermentation, therefore the wines fermented by *S*. *pombe* did not run a serious risk of increasing the levels of histamine or tyramine since they do not need any longer a malolactic fermentation [16].

### Fermentations in stress conditions

Several authors have reported how important is to study yeast performance under stress conditions [70,71]. After performing the stress tests conditions for the selected strains (Table 6) for some parameters described before in literature [34]. We observed that different phenotypic cases were detected for the studied strains in the studied parameters. Other authors have reported before the importance of phenotypic performance in yeasts [72,73]. According to these results strain *S*.*pombe* 936/CECT12774 would develop better than the others *S*.*pombe* under circumstances similar to KCl test and strain JB837/NOTT1 would not be able to perform properly. In the NaCl test strains JB873/NCYC3422, JB4/NCYC683, 935/CECT1376 and 936/CECT12774 performed slightly better than the others *S*.*pombe*. For the ethanol resistance tests, strains JB873/NCYC3422, 935/CECT1376 and 936/CECT12774 could perform better than the others. In the case of KHSO_3_ test, strains JB873/NCYC3422, JB4/NCYC683, 935/CECT1376 and 936/CECT12774 showed to be more resistant. The highest fermenting powers were obtained by strains JB873/NCYC3422, JB4/NCYC683, 935/CECT1376 and 936/CECT12774 among the *S*.*pombe* strains. Even though the values were better for the *S*.*cerevisiae* controls, these differences could be related to the slower developing kinetic describe for *S*.*pombe* than *S*.*cerevisiae* that was observed in the previous trials and reported by other authors [6]. In fact *S*.*pombe* has been described before as highly resistant to some stress conditions [23].

**Table 6:** Final analysis of phenotypic classes for different stressful parameters. Fermentations by selected *S*. *pombe* strains (JB899/Y470, JB917/CBS1057, JB873/NCYC3422, JB4/ NCYC683, JB837/NOTT1 and V1), non-selected *S*. *pombe* strains from the Spanish culture collection (936/CECT12774 and IFI 935/CECT1376) and selected *S*. *cerevisiae* strains (IFI 87/CECT12512 and IFI 88/CECT12513). Number of strains belonging to different phenotypic classes, regarding values of optical density after developing in corrected must with studied stressful parameter (Class 0: A_640_ = 0.1; Class 1: 0.2<A_640_>0.4; Class 2: 0.5<A_640_>1.0; Class 3: A_640_>1.0).

Compounds (mg/L)	899	917	873	4	837	V1	935	936	87	88
KCl (0.75 M)	1	1	2	2	0	1	2	3	3	3
NaCl (1.5 M)	0	0	1	1	0	0	1	1	1	1
Ethanol 6% (% *v/v*)	1	1	2	1	1	1	1	2	3	2
Ethanol 10% (% *v/v*)	0	0	1	0	0	0	1	1	1	1
Ethanol 12% (% *v/v*)	0	0	0	0	0	0	0	0	1	1
Ethanol 14% (% *v/v*)	0	0	0	0	0	0	0	0	0	0
KHSO3 (150 mg/L)	2	2	3	3	1	2	3	3	3	3
KHSO3 (300 mg/L)	1	1	2	2	0	1	2	2	3	3
Fermenting power (Ethanol (% *v/v*))	11.21±0.18b	11.39±0.24b	12.36±0.21c	12.42±0.23c	10.68±0.28a	11.33±0.22b	12.44±0.31c	12.61±26c	13.81±0.29d	13.68±0.33d

Results represent the mean ± SD for three replicates. Means in the same row with the same letter are not significantly different (*p* < 0.05).

### Sensory evaluation

**Fig 4:**
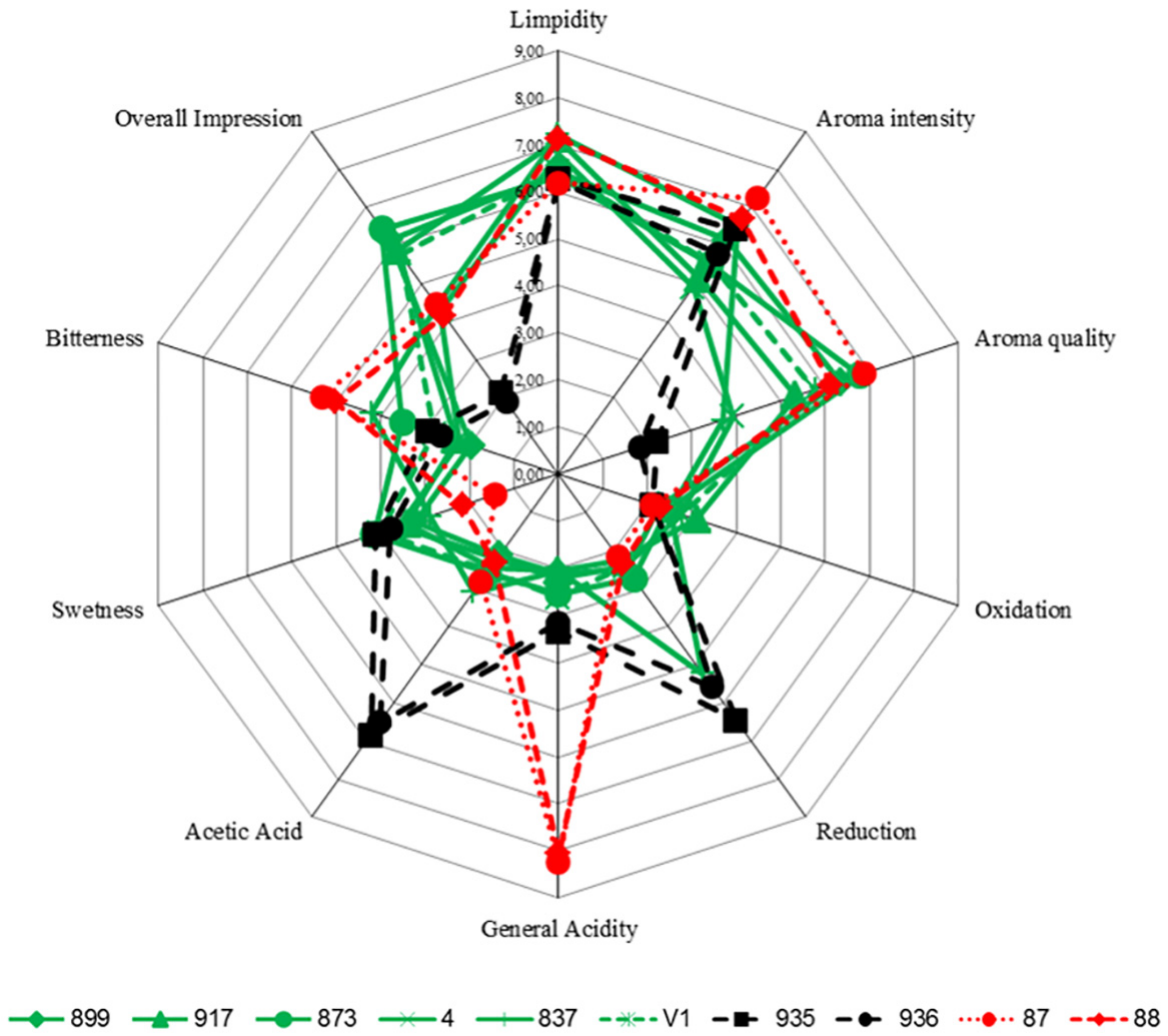
Results of the sensory analysis on fermentation products of selected *S*. *pombe* strains: JB899/Y470, JB873/NCYC3422, JB917/CBS1057, JB4/NCYC683, JB837/NOTT1 and V1; Non selected *S*.*pombe* strains: 936/CECT12774 and IFI935/CECT1376; Selected *S*. *cerevisiae* strains: IFI87/CECT12512 and IFI88/CECT12513.

A sensory evaluation was performed to verify that the selected strains had the potential to produce wines that were pleasant to drink. Fig 4 shows a spider web diagram of the average scores of some olfactory and taste attributes. Large differences in the perception of acidity were recorded; this result agrees with acidity parameters explained above in the previous fermentations. Slight differences were reported regarding to sweetness even though all fermentations depleted successfully all the sugars, this can be explained by differences in acid-sweet balance. Serious faults were reported for non-selected *S*. *pombe* strains IFI935/CECT1376 and 936/CECT12774 regarding to high acetic acid and reduction characters. Fermentations by selected *S*. *pombe* strains JB899/Y470 and JB873/NCYC3422 received the best scores from all tasters while non-selected IFI935/CECT1376 and 936/CECT12774 received the lowest scores, *S*. *cerevisiae* strains IFI87/CECT12512 and IFI88/CECT12513 received moderate scores in overall impression scores related to excessive high acidity for the tasters. These results show that *S*. *pombe* can perform better than *S*.*cerevisiae* under very acidic conditions. The above data show that all fermentations with *S*. *pombe* achieved the main goals related to malic acid deacidification from a very acidic must.

## Conclusions

Among 75 *S*. *pombe* strains tested, we have discovered three strains with highly promising winemaking properties. These strains could be used to produce wines with low levels of malic acid, acetic acid, ethyl carbamate and biogenic amines, and with an appropriate volatile aroma profile. Because these *S*. *pombe* strains produced similar ethanol as the budding yeast strains we examined, this species may well have untapped potential for the producing of ethanol as a fuel (‘bioethanol’).

## Supporting Information

S1 Table First Selection Phase Results. (XLSX)

## References

[1] Su J, Wang T, Wang Y, Li YY, Li H. The use of lactic acid-producing, malic acid-producing, or malic acid-degrading yeast strains for acidity adjustment in the wine industry. Appl Microbiol Biotechnol. 2014; 98: 2395–2413. 10.1007/s00253-014-5508-y 24430209

[2] Jolly NP, Varela C, Pretorius IS. Not your ordinary yeast: Non-Saccharomyces yeasts in wine production uncovered. FEMS Yeast Res. 2014; 14: 215–237. 10.1111/1567-1364.12111 24164726

[3] Benito S, Palomero P, Calderón F, Palmero D, Suárez-Lepe JA. Schizosaccharomyces In: Batt CA, Tortorello ML, editors. Encyclopedia of Food Microbiology (2nd ed.), VOL. 3: Elsevier, Amsterdam, the Netherlands; 2014 pp. 365–370.

[4] Ding S, Zhang Y, Zhang J, Zeng W, Yang Y, Guan J, et al Enhanced deacidification activity in Schizosaccharomyces pombe by genome shuffling. 2015; 32(2): 317–25. 10.1002/yea.3053 25377082

[5] Benito S, Palomero P, Calderón F, Palmero D, Suárez-Lépe JA. Selection of appropriate Schizosaccharomyces strains for winemaking. Food Microbiol. 2014; 42: 218–224. 10.1016/j.fm.2014.03.014 24929740

[6] Benito A, Palomero F, Calderón F, Benito S. Combine Use of Selected Schizosaccharomyces pombe and Lachancea thermotolerans Yeast Strains as an Alternative to the Traditional Malolactic Fermentation in Red Wine Production. Molecules. 2015a; 20: 9510–9523. 26016543 10.3390/molecules20069510 PMC6272599

[7] Palomero F, Morata A, Benito S, Calderón F, Suárez-Lepe JA. New genera of yeasts for over-lees aging of red wine. Food Chem. 2009; 112: 432–441.

[8] Peinado RA, Moreno JJ, Maestre O, Ortega JM, Medina M, Mauricio JC. Gluconic acid consumption in wines by *Schizosaccharomyces pombe* and its effect on the concentrations of major volatile compounds and polyols. J Agric Food Chem. 2004a; 52: 493–497. 14759138 10.1021/jf035030a

[9] Peinado RA, Mauricio JC, Medina M, Moreno JJ. Effect of *Schizosaccharomyces pombe* on aromatic compounds in dry sherry wines containing high levels of gluconic acid. J Agric Food Chem. 2004b; 52: 4529–4534. 15237962 10.1021/jf049853r

[10] Peinado RA, Moreno JJ, Medina M, Mauricio JC. Potential application of a glucose-transport-deficient mutant of *Schizosaccharomyces pombe* for removing gluconic acid from grape must. J. Agric. Food Chem. 2005; 53, 1017–1021. 15713014 10.1021/jf048764b

[11] Peinado RA, Moreno JJ, Maestre O. Removing gluconic acid by using different treatments with a *Schizosaccharomyces pombe* mutant: effect on fermentation by products. Food Chem. 2007; 104: 457–465.

[12] Deák T. Handbook of Food Spoilage Yeasts. 2nd ed CRC Press: Taylor & Francis Group, Boca Raton, USA; 2008 pp. 294–297.

[13] Benito S, Palomero F, Morata A, Calderon F, Palmero D, Suarez-Lepe JA. Physiological features of *Schizosaccharomyces pombe* of interest in making of white wines. Eur Food Res Technol. 2013b; 236: 29–36.

[14] Benito S, Palomero P, Morata A, Calderón F, Suárez-Lépe JA. New applications for *Schizosaccharomyces pombe* in the alcoholic fermentation of red wines. Int J Food Microbiol. 2012; 47: 2101–2108. 10.1016/j.ijfoodmicro.2012.08.007 22921967

[15] Benito S, Palomero P, Gálvez L, Morata A, Calderón F, Palmero D, et al Quality and Composition of Red Wine Fermented with *Schizosaccharomyces pombe* as Sole Fermentative Yeast, and in Mixed and Sequential Fermentations with *Saccharomyces cerevisiae*. Food Technol Biotechnol. 2014c; 52: 376–382.

[16] Benito S. The use of *Schizosaccharomyces* yeast in order to reduce the content of biogenic amines and ethyl carbamate in wines. J Food Process Technol. 2015c; 6(9): 9–10.

[17] Gallander JF. Deacidification of eastern table wines with Schizosaccharomyces pombe. Am J Enol Vitic. 1977; 28: 65–72.

[18] Snow PG, Gallander JF. Deacidification of white table wines trough partial fermentation with Schizosaccharomyces pombe. A J Enol Vitic. 1979; 30: 45–48.

[19] Unterholzner O, Aurich M, Platter K, Geschmacks und Geruchsfehler bei Rotweinen verursacht durch Schizosaccharomyces pombe L. Mitteilungen Klosterneuburg, Rebe und Wein, Obstbau und Früchteverwertung. 1988; 38: 66–70.

[20] Yokotsuka K, Otaki A, Naitoh H. Controlled Simultaneous deacidification and alcohol fermentation of a high acid grape must using two immobilized yeasts, Schizosaccharomyces pombe and Saccharomyces cerevisiae. A. J. Enol. Vit. 1993; 44: 371–377.

[21] Pitt JI, Hocking AD. Fungi and Food Spoilage. 2nd ed An Aspen Publication: Gaithersburg; 1999 pp. 459–460.

[22] http://www.proenol.com/files/editorials/ProMalic_FT204-09_PT.pdf?u=. Accessed 18 September 2015.

[23] Benito S, Gálvez L, Palomero F, Calderón F, Morata A, Suárez-Lepe JA. *Schizosaccharomyces* selective differential media. Afr. J. Microbiol. Res. 2013; 7: 3026–3036.

[24] Jeffares DC, Rallis C, Rieux A, Speed A, Převorovský M, Mourier T, et al The genomic and phenotypic diversity of *Schizosaccharomyces pombe*. Nat Genet. 2015; 47: 235–241. 10.1038/ng.3215 25665008 PMC4645456

[25] Bai FY. State Key Laboratory of Mycology, http://www.mycolab.org.cn/templates/T_second_EN/index.aspx?nodeid=430. Accessed 18 September 2015.

[26] Pretorius IS, Bauer FF, Meeting the consumer challenge through genetically customized wine-yeast strains. Trends in Biotechnol. 2002; 20: 426–432. 10.1016/s0167-7799(02)02049-8 12220905

[27] Husnik JL, Volschenk H, Bauer J. Metabolic engineering of malolactic wine yeast. Metabol Eng. 2006: 8: 315–323. 10.1016/j.ymben.2006.02.003 16621641

[28] Husnik JL, Delaquis PJ, Cliff MA. Am J Enol Vitic. 2007; 58:42–52

[29] Liu YL, Li H. Integrated Expression of the *Oenococcus oeni* mleA Gene in *Saccharomyces cerevisiae*. Agr Sci China. 2009; 8: 821–827.

[30] http://www.magrama.gob.es/es/calidad-y-evaluacion-ambiental/temas/biotecnologia/organismos-modificados-geneticamente-omg-/legislacion-general/legislacion_europea.aspx. Accessed 18 September 2015.

[31] Vaughnan-Martini A, Martini A. Determination of ethanol production In: Kurtzman CP, Fell JW, editors. The Yeast. A Taxonomic Study. Elsevier: Amsterdam, Netherlands; 1999 p. 107.

[32] http://www.biosystems.es/products/ENOLOGY/Reactivos_EnologY/ENZIM%c3%81TICOS. Accessed 18 September 2015.

[33] http://shop.gabsystem.com/b2c/producto/1010004/1/ebulliometro-gab. Accessed 18 September 2015.

[34] Mendes I, Franco-Duarte R, Umek L, Fonseca E, Drumonde-Neves J, Dequin S, et al Computational Models for Prediction of Yeast Strain Potential for Winemaking from Phenotypic Profiles. PLOS One.2013, 8(7): 1–10. 10.1371/journal.pone.0066523 PMC3713011 23874393

[35] Vaughan-Martini A, Martín A. Determination of ethanol production In: The yeasts. A taxonomic study. Eds. Kurtzman CP, Fell JW, Elsevier 1998; p 107.

[36] http://www.oiv.int/oiv/files/3%20-%20Resolutions/EN/2012/OIV-OENO%20370-2012.pdf.

[37] Fleet GH. Wine yeasts for the future. FEMS Yeast Res. 2008; 8(7): 979–995. 10.1111/j.1567-1364.2008.00427.x 18793201

[38] Silva S, Ramón-Portugal F, Andrade P, Texera M, Strehaino P. Malic acid consumption by dry immobilized cells of Schizosaccharomyces pombe. A J Enol Vitic. 2003; 54: 50–55.

[39] Rodriguez SB, Thornton RJ. Factors influencing the utilisation of L-malate by yeasts. FEMS Microbiol Lett. 1990; 72: 17–22. 10.1016/0378-1097(90)90337-p 2283035

[40] Thornton RJ, Rodriguez SB. Deacidification of red and white wines by a mutant of Schizosaccharomyces malidevorans under commercial winemaking conditions. Food Microbiol. 1996; 13: 475–482.

[41] Redzepovic S, Orlic S, Majdak A, Kozima B, Volschenk H, Viljoen-Bloom M. Differential malic acid degradation by selected strains of *Saccharomyces* during alcoholic fermentation. Int J Food Microbiol. 2003; 83: 49–61. 12672592 10.1016/s0168-1605(02)00320-3

[42] Belda I, Navascués E, Marquina D, Santos A, Calderón F, Benito S. Dynamic analysis of physiological properties of *Torulaspora delbrueckii* in wine fermentations and its incidence on wine quality. Appl Microbiol Biotechnol. 2015; 99(4): 1911–22. 10.1007/s00253-014-6197-2 25408314

[43] Benito S, Hofmann T, Laier M, Lochbühler BC, Schüttler A, Ebert K, et al Effect on quality and composition of Riesling wines fermented by sequential inoculation with non-*Saccharomyces* and *Saccharomyces cerevisiae*. Eur Food Res Technol. 2015b; 241(5):707–717.

[44] Jolly NP, Augustyn OPH, Pretorius IS. The Role and Use of Non-Saccharomyces Yeasts in Wine Production. S Afr J Enol Vitic. 2006; 27: 15–39.

[45] Kutyna DR, Varela C, Henschke PA, Chambers PJ, Stanley GA. Microbiological approaches to lowering ethanol concentration in wine. Trends Food Sci. Technol. 2010; 21: 293–302.

[46] Gobbi M, Comitini F, Domizio P, Romani C, Lencioni L, Mannazzu I, et al Fermentative aptitude of non-*Saccharomyces* wine yeast for reduction in the ethanol content in wine. Eur Food Res Technol. 2014; 239(1): 41–48.

[47] Contreras A, Hidalgo C, Henschke PA, Chambers PJ, Curtin C, Varela C. Evaluation of Non-Saccharomyces Yeasts for the Reduction of Alcohol Content in Wine. Appl Environ Microb. 2014; 80: 1670–1678. 10.1128/AEM.03780-13 PMC3957604 24375129

[48] Merico A, Sulo P, Piskur J, Compagno C. Fermentative lifestyle in yeasts belonging to the Saccharomyces complex. FEBS J. 2007; 274: 976–989. 17239085 10.1111/j.1742-4658.2007.05645.x

[49] Bely M, Stoeckle P, Masneuf-Pomarède I, Dubourdieu D. Impact of mixed *Torulaspora delbrueckii*–*Saccharomyces cerevisiae* culture on high-sugar fermentation. Int J Food Microbiol. 2008; 122: 312–320. 10.1016/j.ijfoodmicro.2007.12.023 18262301

[50] Contreras A, Curtin C, Varela C. Yeast population dynamics reveal a potential 'collaboration' between Metschnikowia pulcherrima and Saccharomyces uvarum for the production of reduced alcohol wines during Shiraz fermentation. Appl Microbiol Biotechnol. 2015; 99(4): 1885–95. 10.1007/s00253-014-6193-6 25388943

[51] Morales P, Rojas V, Quirós M, González R. The impact of oxygen on the final alcohol content of wine fermented by a mixed starter culture. Appl Microbiol Biotechnol. 2015; 99(9): 3993–4003. 10.1007/s00253-014-6321-3 25582558 PMC4428804

[52] Lubbers MW, Rodriguez SB, Honey NK, Thornton RJ. Purification and characterization of urease from *Schizosaccharomyces pombe*. Canad J Microbiol. 1996; 42: 132–40. 8742356 10.1139/m96-021

[53] Xia C, Hong M, YunFeng Z, Ning WY. Research progress on toxicity and contamination of ethyl carbamate in fermented foods. J Food Safety Quality. 2014; 5(9): 2617–262.

[54] Benito A, Calderón F, Palomero F, Benito S. Quality and Composition of Airen Wines Fermented by Sequential Inoculation of *Lachancea thermotolerans* and *Saccharomyces cerevisiae*. Food Technol. Biotechnol. Accepted 2015 10.17113/ftb.54.02.16.4220 PMC5105610 27904403

[55] Clemente-Jiménez JF, Mingorance-Cazorla L, Martínez-Rodríguez S, Las Heras-Vázquez FJ, Rodríguez-Vico F. Molecular characterization and oenological properties of wine yeast isolated during spontaneous fermentation of six varieties of grape must. Food Microbiol. 2004; 21: 149–155.

[56] Parapouli M, Hatziloukas E, Drainas C, Perisynakis A. The effect of Debina grapevine indigenous yeast strains of *Metschnikowia* and *Saccharomyces* on wine flavour. J Ind Microbiol Biot. 2010; 37: 85–93. 10.1007/s10295-009-0651-7 19859752

[57] Gobbi M, Comitini F, Domizio P, Romani C, Lencioni L, Mannazzu I, et al Lachancea thermotolerans and Saccharomyces cerevisiae in simultaneous and sequential co-fermentation: a strategy to enhance acidity and improve the overall quality of wine. Food Microbiol. 2013; 33: 271–281. 10.1016/j.fm.2012.10.004 23200661

[58] Romano P, Suzzi G, Comi G, Zironi R. Higher alcohol and acetic acid production by apiculate wine yeasts. J Appl Bact. 1992; 73: 126–130.

[59] Zironi R, Romano P, Suzzi G, Battistutta F. Comi G. Volatile metabolites produced in wine by mixed and sequential cultures of *Hanseniaspora guilliermondii* or *Kloeckera apiculata* and *Saccharomyces cerevisiae*. Biotechnol Lett. 1993; 15: 235–238.

[60] Romano P, Suzzi G. Higher alcohol and acetoin production by *Zygosaccharomyces* wine yeasts. J Appl Bacteriol. 1993; 75: 541–545.

[61] Maynard LS, Schenker VJ. Monoamine-oxidase inhibition by ethanol in vitro. Nature. 1996; 196: 575–576. 10.1038/196575a0 13934158

[62] Kanny G, Gerbaux V, Olszewski A, Frémont S, Empereur F, Nabet F, et al No correlation between wine intolerance and histamine content of wine. J Allergy Clin Inmmun. 2001; 107: 375–378. 10.1067/mai.2001.112122 11174207

[63] Jansen SC, van Dusseldorp M, Bottema KC, Dubois AE. Intolerance to dietary biogenic amines: A review. Ann Allerg Asthama Im. 2003; 91: 233–240. 10.1016/S1081-1206(10)63523-5 14533654

[64] Moreno-Arribas MV, Polo MC. Occurrence of lactic acid bacteria and biogenic amines in biologically aged wines. Food Microbiol. 2008; 25: 875–881. 10.1016/j.fm.2008.05.004 18721676

[65] Lehtonen P. Determination of amines and amino acids in wine: A review. Am J Enol Vitic. 1996; 47(2): 127–133.

[66] Medina K, Boido E, Fariña L, Gioia O, Gomez ME, Barquet M, et al Increased flavour diversity of Chardonnay wines by spontaneous fermentation and co-fermentation with *Hanseniaspora vineae*. Food Chem. 2013, 141(3):2513–21. 10.1016/j.foodchem.2013.04.056 23870989

[67] Alcaide-Hidalgo JM, Moreno-Arribas MV, Martín-Álvarez PJ, Polo MC. Influence of malolactic fermentation, postfermentative treatments and ageing with lees on nitrogen compounds of red wines. Food Chem. 2007; 103: 572–581. 10.1016/j.foodchem.2007.12.040 26054276

[68] Smit AY, du Toit M. Evaluating the Influence of Malolactic Fermentation Inoculation Practices and Ageing on Lees on Biogenic Amine Production in Wine. Food Bioprocess Technol. 2013; 6: 198–206.

[69] Sumby KM, Grbin PR, Jiranek V. Implications of new research and technologies for malolactic fermentation in wine. Appl Microbiol Biotechnol. 2014; 98: 8111–8132. 10.1007/s00253-014-5976-0 25142694

[70] Zheng YL, Wang SA. Stress Tolerance Variations in Saccharomyces cerevisiae Strains from Diverse Ecological Sources and Geographical Locations. PLOS One. 2015 10.1371/journal.pone.0133889 PMC4526645 26244846

[71] Bonciani T, Solieri L, De Vero L, Giudici P. Improved wine yeasts by direct mating and selection under stressful fermentative conditions. Eur Food Res Technol. 2015, Accepted 10.1007/s00217-015-2596-6

[72] Camarasa C, Sanchez I, Brial P, Bigey F, Dequin S. Phenotypic Landscape of Saccharomyces cerevisiae during Wine Fermentation: Evidence of Origin-Dependent Metabolic Traits. PLOS One.2011, 6(9): 1–10. 10.1371/journal.pone.0025147 PMC3174997 21949874

[73] Hyma KE, Saerens SM, Verstrepen KJ, Fay JC. Divergence in wine characteristics produced by wild and domesticated strains of *Saccharomyces cerevisiae*. FEMS Yeast Res. 2011, 11(7):540–51. 10.1111/j.1567-1364.2011.00746.x 22093681 PMC3262967

